# Alginate Films Enriched in Raspberry and/or Black Currant Seed Oils as Active Food Packaging

**DOI:** 10.3390/molecules29092012

**Published:** 2024-04-27

**Authors:** Jolanta Kowalonek, Bogna Łukomska, Olga Łukomska, Natalia Stachowiak-Trojanowska

**Affiliations:** Department of Biomedical and Polymer Chemistry, Faculty of Chemistry, Nicolaus Copernicus University in Torun, Gagarina St. 7, 87-100 Torun, Poland; bognalukomska@abs.umk.pl (B.Ł.); olgalukomska@abs.umk.pl (O.Ł.); naat.staa@gmail.com (N.S.-T.)

**Keywords:** alginate film, raspberry oil, black currant oil, infrared spectroscopy

## Abstract

Alginate films plasticized with glycerol and enriched in raspberry and/or black currant seed oils were prepared via casting solution techniques. The intention was to create active films for food packaging where antioxidants in a film would deactivate oxidants in a packed product or its surroundings, improving conditions inside packaging and extending the shelf life of such a product. The prepared materials were characterized by physicochemical, spectroscopic, mechanical, water vapor transmission (WVTR), and antioxidant activity analysis. Infrared spectra of the alginate films with oils were similar to those without the additive; the band with a maximum at about 1740 cm^−1^ stood out. The prepared materials with oils were thicker, contained less water, were more yellow, and were less permeable to water vapor. Moreover, the presence of the oil in the films resulted in a slightly lower Young’s modulus and lower stress at break values but higher strain at break. The antioxidant capacity of raspberry seed oil itself was about five times higher than that of black currant seed oil, and a similar trend was noticed for films modified with these oils. The results indicated that both oils could be used as active substances with antioxidant properties in food packaging.

## 1. Introduction

Many contemporary goods are made of plastics, or if they are not entirely made of plastic, they have plastic elements. This is because synthetic polymers have good mechanical properties, are easy to process, and are cheap. Thus, plastics produced from synthetic polymers are ubiquitous in our lives. One-third of their usage is in packaging films [1]. After usage, all the plastics end up in landfills, degrading over the years and threatening the natural environment. The topic of packaging is still vital in terms of reducing waste. This is why there is a need for biopolymer substitutes that fulfill the requirements for packaging, such as having good mechanical, optical, and barrier properties. One of the solutions is the production of edible films and coatings. Such packaging is intended to protect perishable food, thus reducing food waste and minimizing waste from packaging [1,2,3,4].

Such natural polymer substitutes encompass biopolymers such as polysaccharides and proteins, which are degradable and occur abundantly in nature [5]. The disadvantages of neat polysaccharides are their poor mechanical properties, stiffness, and fragility, but these drawbacks can be overcome by adding a plasticizer to the film [1,4]. Moreover, adding suitable active compounds to the packaging can result in obtaining a film with antioxidant and antibacterial properties being obtained, prolonging the shelf life of packed food. One can also monitor food quality by producing intelligent films sensitive to pH, temperature, or color changes [3,6,7,8].

Using glycerol as a plasticizer in polysaccharide films increases the moisture content in these films, which is a disadvantage because the growth of fungi and mold is very likely in wet environments. The introduction of oils in the films reduces their moisture content [1].

Among biopolymers, alginate has good film-forming properties; it is biodegradable and non-toxic, making it suitable for eco-friendly packaging.

Alginate is a naturally occurring polysaccharide that can be isolated from the cell walls of brown seaweeds. This biopolymer can exist as an alginic acid, which is insoluble in water, or transformed into a water-soluble sodium alginate. Alginate is a polysaccharide comprising α-l-guluronate (G) and β-d-mannuronate (M) units. These units can be linked in different ways and form sequences of only mannuronate units (M-blocks), guluronate (G-blocks), or alternating units (MG-blocks). The unit arrangements have consequences; M units are connected by β (1–4) glycosidic bonds forming a linear flexible structure, whereas G units are linked by α (1–4) glycosidic bonds to build a tighter and more rigid structure with the ability to bind divalent cations and form cross-linked networks [1,4,5].

Alginate has found countless applications, for instance, in the food industry (as a thickening agent, emulsifier, stabilizer, edible food packaging), biomedicine and pharmaceutical industry (encapsulation of drugs, wound dressing, binders and coating for tablets), and textile printing [1,9] 

Seeds of raspberry and black currant are a by-product of fruit processing. The seeds are a valuable source of nutrients and can be used for oil production. Edible oils are mainly composed of triglycerides and free fatty acids as a result of triglycerides’ hydrolysis. Raspberry seed oil (RSO) contains mainly the following fatty acids: linoleic, α-linolenic, oleic, and palmitic [10,11,12], listed in order of occurrence in an immense amount. Moreover, there are the phenolic acids (gallic, caffeic, ferulic) [10], carotenoids that give the oil a yellow color, tocopherols and tocotrienols [10,11], and a small amount of chlorophyll in its composition.

The composition of black currant seed oil (BCSO) differs from that of raspberry seed oil. Besides the fatty acids mentioned above for raspberry seed oil, γ-linolenic acid is present in black currant seed oil [11]. Moreover, α-, γ-, and δ-tocopherols, phenolic compounds, and carotenoids have been found in BCSO [13,14]. This oil is dark green, indicating that the chlorophyll pigment is dominant. 

This study aimed to investigate the effect of raspberry seed oil and/or blackcurrant seed oil on alginate films’ physicochemical and antioxidant properties in order to use them as active food packaging films. There is no information on biopolymer films or coatings containing raspberry or black currant seed oils as an active component. 

## 2. Results

### 2.1. ATR-FTIR Spectroscopy Results

**Figure 1:**
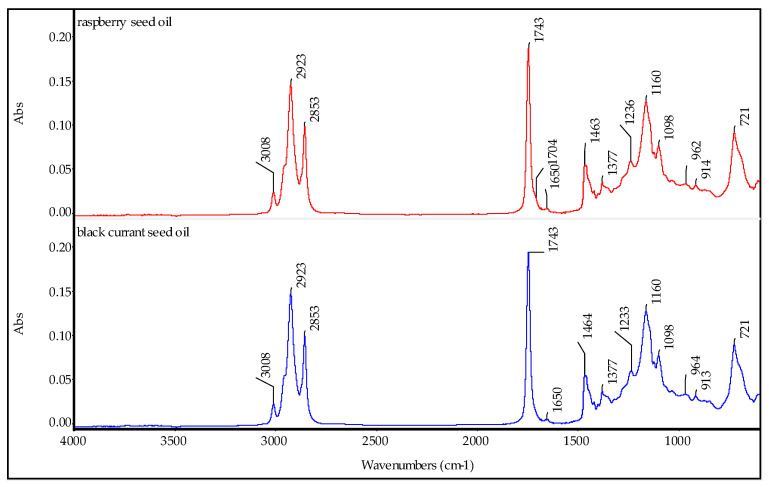
ATR-FTIR spectra of black currant seed oil and raspberry seed oil.

ATR-FTIR spectra of the applied fruit seed oils are presented in Figure 1. The absorption bands’ maxima and the appropriate bond vibrations assigned to them are gathered in Table 1. As can be seen, both spectra are similar. Only slight changes in band intensities and positions were detected. Infrared spectra of the studied oils showed the presence of functional groups characteristic of triacylglycerols and fatty acids. Based on the spectra, one can analyze the saturated to unsaturated fatty acids’ ratio. According to [10,15], the ratio of band intensities at 3008 cm^−1^/2923 cm^−1^ reflecting the =C−H bond vibrations in *cis* structures to −C−H bond vibrations can be treated as a measure of the degree of unsaturation. Thus, this ratio was 0.14 for the BCSO and 0.15 for the RSO. The higher value means a higher content of unsaturated fatty acids in oil. Our values were much lower when compared with those obtained by Rajagukguk et al. [10], who tested commercially and laboratory-produced raspberry seed oil, and the ratios of 3011 cm^−1^/2925 cm^−1^ for the tested samples were in the range of 0.17–0.24. Next, vibrations of C=O in ester were observed at 1743 cm^−1^, and the presence of the small shoulder at 1705 cm^−1^ observed in the RSO spectrum confirmed the presence of free fatty acids [16] in this oil, not seen in the spectrum of BCSO. Other bands giving information on the oil composition were bands at 964 cm^−1^, 984 cm^−1^, and 721 cm^−1^ assigned to =CH modes in *trans* nonconjugated, *trans* conjugated, and *cis* isomers, respectively. The *trans* isomers can appear in the sample due to their *cis* counterpart isomerization induced by heat or oxidation. The bands at 984 cm^−1^ and in the region of O−H bond vibrations were not detected, which suggested that the traces of *trans* isomers formed while pressing the oils [10].

**Figure 2:**
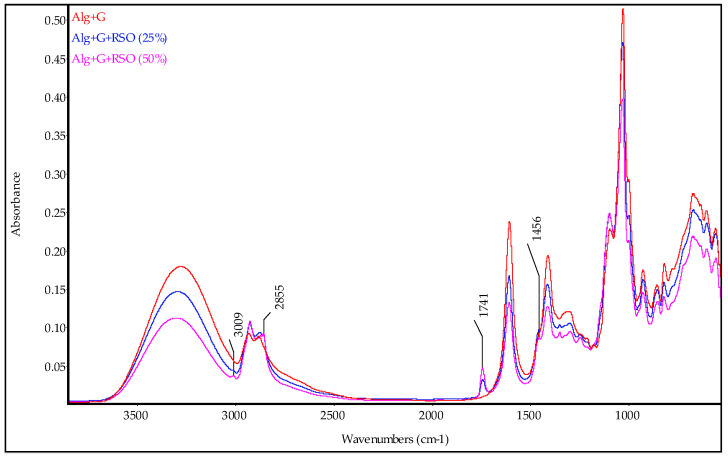
ATR-FTIR spectra of alginate films without and with different contents of raspberry seed oil. In figure, the bands coming from oil are marked.

Figure 2 shows the ATR-FTIR spectra of the plasticized alginate films with and without raspberry seed oil. Only bands typical of oil were marked in Figure 2 to see better the differences between the biopolymer spectra without oil and with oil. The spectrum of alginate film without the oil was described, but the absorption bands were not marked in order for the bands coming from functional groups of the added oil in alginate film to be more visible. The infrared spectrum of alginate film (red line) exhibited a broad absorption band with a maximum at 3278 cm^−1^ assigned to O−H stretching vibrations. The bands at 2930 cm^−1^ and 2880 cm^−1^ were attributed to C−H asymmetric and symmetric stretching, respectively. Next, 1604 cm^−1^ and 1408 cm^−1^ bands indicated COO^−^ asymmetric and symmetric stretching, respectively. The less intense band at 1297 cm^−1^ was assigned to C−H wagging vibrations. An intense band with three maxima at 1094 cm^−1^, 1026 cm^−1^, and 997 cm^−1^ was a result of stretching vibrations of C−O−C in glycosidic linkages (the first and the second maxima) and asymmetric stretching of C−O in C−O−H (the third maximum). The following bands are characteristics of glycerol. The band at 925 cm^−1^ belonged to O−H deformation, and 853 cm^−1^ was associated with C−O symmetric stretching vibrations. The low-intensity band at 816 cm^−1^ was attributed to out-of-plane stretching of −C−H in polysaccharides.

When raspberry seed oil was introduced into the biopolymer film, the spectra of the alginate films with oil revealed some changes, such as the appearance of bands typical of oil, the band shifting, and a decreasing or increasing in intensity (Figure 2). Thus, the low-intensity bands at 3009 cm^−1^, 2855 cm^−1^, and 1741 cm^−1^, and shoulder at 1456 cm^−1^ in the alginate spectrum proved the presence of oil in the sample. Moreover, most bands of sodium alginate moved slightly towards higher wavenumbers and decreased their intensity; only the bands at 1094 cm^−1^ and 2924 cm^−1^ increased their intensity. Generally, based on the infrared spectra, one can infer weak interactions between macromolecules and oils resulting from the hydrophobic nature of oils. 

ATR-FTIR spectra of alginate films with black currant seed oil and the oil mixture were similar to those obtained and presented for alginate films with raspberry seed oil.

### 2.2. Thickness, Opacity, Moisture Content, and Water Vapor Transmission Rate (WVTR) Results

Table 2 shows the thicknesses of the studied films. The thickness influences the other tested quantities, such as moisture content, WVTR, and mechanical properties. It is seen that the addition of oil to the biopolymer film increased film thickness; the effect is apparent for samples with a higher content of the oil. The thickness increased because the distances between alginate macromolecules increased, and their interactions weakened due to the presence of oils. Similar results were obtained by other researchers [22,23]. Moreover, films with black currant seed oil were thinner than those with raspberry seed oil because of the different compositions of the oils. As the conditions of preparing and drying films influence film thickness, the samples were prepared and dried under the same temperature and time conditions to be comparable.

Another feature of packaging is its opacity. Packaging’s opacity or transparency is an important feature determining how a consumer sees the product. Opacity is the ability to block light. This experiment defined this parameter as a ratio of absorbance at 600 nm and film thickness [24]. Low values of opacity indicated good film transparency. Alginate film with glycerol was characterized by the lowest opacity value, which increased much after adding oils to the alginate films. This was due to the forming emulsion and immiscibility of the hydrophilic biopolymer and hydrophobic oils. The highest opacity was for films with an oil mixture, and the lowest was for films with BCSO.

Moisture can facilitate the growth of bacteria and molds that cause food spoilage. On the other hand, moisture also prevents food from drying out. The highest moisture content was found in the neat biopolymer films due to glycerol being capable of binding water. The addition of oil resulted in a decrease in moisture content due to the addition of hydrophobic substances. This effect was more pronounced in the films with a higher oil content. Moreover, the moisture content in films with RSO was higher than the rest of the films, resulting from their different oil compositions. 

The water vapor transmission rate determines the moisture barrier of the film to prevent the product from spoilage. The barrier properties of the films improved when oils were introduced to the tested films due to the presence of hydrophobic ingredients in the films. The distinguishing WVTR values were found for two films: the one with a higher content of oil mixture and the other with a higher content of RSO. 

The studies of antioxidant capacity, carried out by the DPPH method, revealed that the AC value of raspberry seed oil was significantly higher than that of black currant seed oil; namely, the AC value was 922 ± 63 (μmol Trolox/100 g sample) for raspberry seed oil and 162 ± 10 (μmol Trolox/100 g sample) for black currant seed oil. Matysiak-Żurowska et al. found that raspberry seed oil had a higher antioxidant capacity than black currant seed oil, tested with the DPPH and ABTS methods; however, the differences between the AC values were not so pronounced [11]. Both methods revealed a positive correlation with tocopherol contents. In the case of carotenoids, AC by the DPPH method showed a weak negative correlation with carotenoids content, whereas ABTS revealed a positive correlation with carotenoids content. Trela et al. tested vegetable and fruit seed oils regarding the influence of tocochromanols on DPPH scavenging [25]. The results indicated a strong positive correlation between DPPH scavenging and tocochromanols’ content in the tested oil. Many compounds, such as tocopherols, phenolics, and carotenoids, can react with DPPH and consequently influence AC. In this experiment, the films with RSO showed much higher ACs than those with BCSO; the mentioned publications also demonstrated such a relationship [11,25]. Moreover, films with a mixture of oils exhibited lower antioxidant capacities than would result from the additive rule, suggesting a mutual deactivating effect of the oil components on each other in a mixture. 

### 2.3. Mechanical Properties

The mechanical properties of the packaging are essential from a practical point of view. Packaging should have good tensile strength and flexibility to endure forces acting on them while products are transported and stored. Table 3 shows the Young’s modulus, stress, and strain at the break of the tested films. It is seen that the addition of oils resulted in a decrease in Young’s modulus and the tensile strength with a simultaneous increase in the strain at break, which suggested the plasticizing effect of the added oils due to the increase in free volume and macromolecules’ mobility, which was a result of the reduction in hydrogen bonding and weakened interactions between alginate macromolecules. The more oil is added, the better the plasticizing effect will be. Moreover, BCSO exhibited a slightly more significant plasticizing impact due to the different oil compositions.

### 2.4. Antibacterial Activity

Antibacterial properties of the studied films were tested against four strains of bacteria: *Escherichia coli*, *Staphylococcus aureus*, *Bacillus Subtilis*, *and Pseudomonas aeruginosa*. Only films with a higher content of a given oil were tested. The alginate film with glycerol was not tested this time regarding antibacterial activity due to it lacking such an effect as demonstrated in our previous research [26] and the works of other researchers [27].

**Figure 3:**
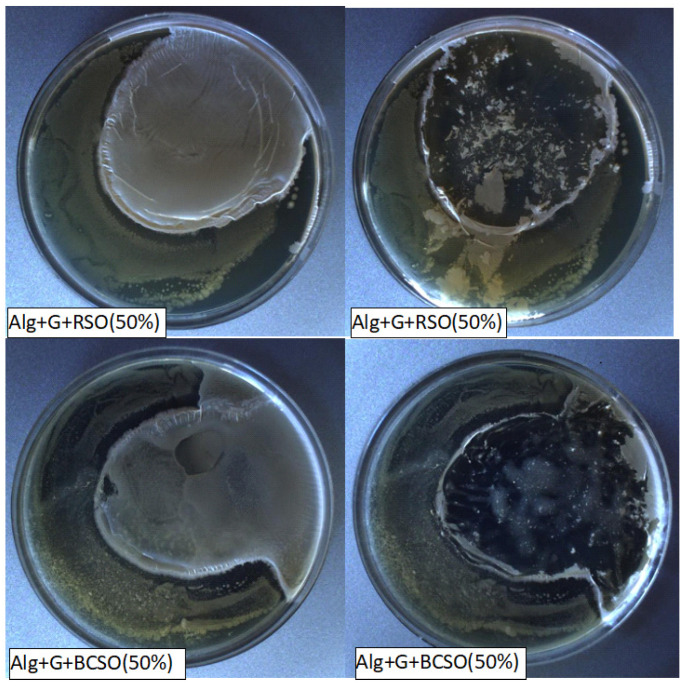
Growth of *Escherichia coli* on agar medium with the studied films (**left side**) and after removing the films (**right side**).

Analyzing the antibacterial activity of the films against standard strains of bacteria, it was found that the tested films had no antibacterial activity against *Pseudomonas aeruginosa* and *Staphylococcus aureus* (Figure S1) and very weak activity against *Escherichia coli* (Figure 3). In contrast, the growth of *Bacillus Subtilis* (Figure S1) was partially inhibited; only slight turbidity can be seen in the photos after removing the films. Turbidity means multiplied bacteria, which are especially visible in the case of *Pseudomonas aeruginosa* and *Staphylococcus aureus* (Figure S1). There was no difference between the actions of the studied oils against mentioned bacteria. Moreover, this weak antimicrobial activity was observed for Gram-negative (*E. coli*) and Gram-positive microorganisms (*B. subtilis*), suggesting no relation between antimicrobial effect and the structure of the cell walls of bacteria.

In the literature, information about the antibacterial effect of oils is ambiguous. Studies on alginate/castor oil edible films revealed antibacterial properties against Gram-positive bacteria (*S. Aureus*, *B. Subtilis*) but no effect was observed for Gram-negative bacteria (*E. coli*, *S. typhi*) [27]. Other research showed weaker antibacterial properties of virgin olive oil and grape seed oil than savory essential oil. Moreover, their activity against Gram-negative bacteria was more significant than against Gram-positive [22]. Fangfang et al. showed that starch-based films with virgin coconut oil (VCO) had antimicrobial properties. The antimicrobial effect was more intens against Gram-positive bacteria (*S. aureus* and *L. monocytogenes*) than Gram-negative bacteria (*E. coli*). This behavior was explained by the ability of triglycerides in VCO to destroy bacterial cell walls, which was easier in the case of Gram-positive than Gram-negative microorganisms due to the different structures of the cell walls of bacteria [28].

## 3. Discussion

The incorporation of fruit seed oils into the alginate film altered its properties. The tensile strength of modified films decreased, while strain at break increased. The thickness increased, and the moisture content decreased. The same trends were observed for the gelatin and sodium alginate films containing grape seed oil nanoemulsion (GSO-NE) [22]. Moreover, the sample with the highest GSO-NE content had antibacterial properties, whereas the films in our work showed very weak or lack antibacterial activities. In other work, the authors found sodium alginate films with rapeseed, hazelnut, or coconut oil were thicker and more opaque than the neat sodium alginate film [24]. At the same time, moisture content was lower for alginate films with the mentioned oils [24]. These results were similar to our findings. Other studies of alginate film with castor oil showed better mechanical properties than neat alginate films due to the formation of hydrogen bonds and electrostatic interactions between alginate and castor oil [27]. A reduction in vapor permeability and antibacterial activity against Gram-positive bacteria was observed. Colivet et al. [29] studied cassava starch films with watermelon seed oil. The films with watermelon seed oil were more flexible but did not show significant changes in moisture content and barrier properties, which was explained by the distribution of oil particles in the polymeric film and the starch’s hydrophilic nature. Khah et al. studied films made of pectin and gelatin containing olive oil and grape seed oil [30]. They demonstrated a decrease in the stiffness of the films with oils, and the type of oil was also important, which was explained by their different oil compositions, the droplet size of emulsion, and interactions with biopolymers. Moreover, water vapor permeability was reduced in the case of films with oils; a slightly higher effect was observed for films with olive oil. The authors found that samples with oil showed weak antibacterial activity. 

Generally, our findings for films with fruit seed oils were consistent with the results obtained by other researchers; however, our films did not have satisfactory antibacterial activity.

## 4. Materials and Methods

### 4.1. Materials

**Figure 4:**
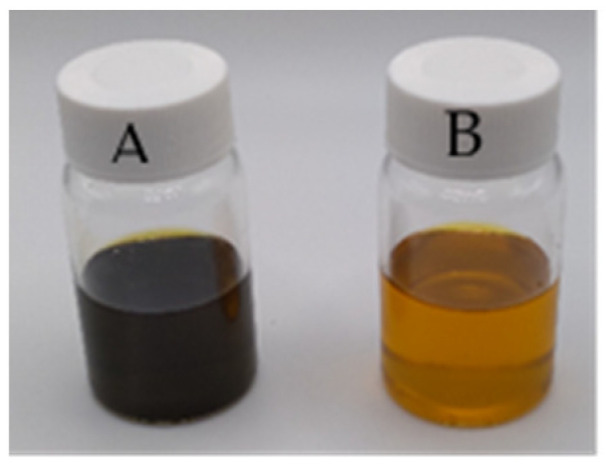
The picture of black currant seed oil (**A**) and raspberry seed oil (**B**).

Sodium alginate (ALG) was acquired from Büchi Labortechnik AG (Flawil, Switzerland); glycerol (G), methanol (pure for analysis), and ethanol (96%, pure for analysis) were bought from Avantor Performance Materials Poland S.A. (Gliwice, Poland); raspberry seed oil (*Rubus idaeus*) (RSO) and black currant seed oil (*Ribes nigrum*) (BCSO) were purchased from Etja S.C. (Elbląg, Poland) (Figure 4); a surfactant TWEEN 80 was bought from Greenaction (Kielce, Poland); and 2,2-diphenyl-1-picrylhydrazyl (DPPH, 95%) and 6-hydroxy-2,5,7,8-tetramethylchromane-2-carboxylic acid (Trolox, 97%) were supplied by Sigma Aldrich (Poznań, Poland).

### 4.2. Determination of Physicochemical Parameters of the Tested Oils

The tested oils were cold-pressed and unrefined. The acid value (AV), iodine value (IV), and peroxide value (PV) were determined (Table 4). The acid value is a measure of free fatty acid, which determines the degree of hydrolysis of lipids. It is expressed as a milligram of KOH needed to neutralize fatty acid in 1 g of the sample, determined according to norm PN-ISO 660:1998 [31]. 

The iodine value is a measure of the degree of unsaturation of lipids. It is expressed in grams of I_2_ consumed by a fat sample of weight 100 g. It was determined from the equation [32,33]:

n_D_^25^ = 1.45795 + 0.0001164·IV

where: n_D_^25^—refractive index of tested oil, IV—iodine value.

Peroxide value (PV) is a measure of peroxide content in the fat sample. It reflects the oxidation (rancidity) of fat. PV is expressed as a milliequivalent of oxygen per 1000 g of fat. PV was determined according to norm PN-ISO 3960:1996 [34].

The obtained results (Table 4) showed higher AVs and PVs of these oils than the common edible oils, which are typical of unrefined oils [35]. A high IV indicates a large amount of unsaturated compounds.

### 4.3. Determination of Sodium Alginate Molecular Weight

The molecular weight of sodium alginate was determined using the viscometric method. First, 0.1 M NaCl aqueous solution was prepared in which sodium alginate was dissolved, forming a 0.1% (*w*/*v*) solution relative to biopolymer. Then, the solution was filtered with funnel G1 and placed in the Ubbelohde viscometer (type 532 10, K constant 0.01 mm^2^/s) immersed in water bath CT72/P (Si Analytics, Mainz, Germany) at 25 °C. The flow times of the polymer solutions of different concentrations were measured with the Viscoclock Plus (Si Analytics, Mainz, Germany) device. The limiting viscosity number, [η], was obtained from Huggins and Kraemer plots. The viscosity-average molecular weight was calculated from the Mark–Houwink–Sakurada equation, $\left[\eta \right]=K\bar{M_{v}^{a}}$, and was equal to $\bar{M_{v}}=53000$ for K = 0.0178 cm^3^/g and a = 1 [26].

### 4.4. Film Preparation

**Figure 5:**
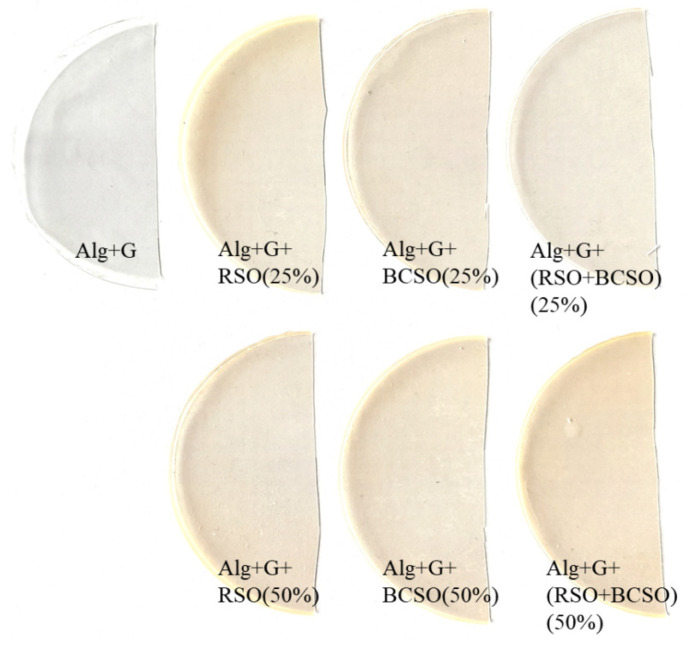
The pictures of the prepared films.

An alginate sodium aqueous solution of 2% (*w*/*v*) was prepared. To this solution, glycerol of the amount of 2.5% (*w*/*v*) was added. Separately, mixtures of oils with surfactant (Tween 80) were prepared in a ratio of 1:1. Next, fixed amounts of the mixture of oil with surfactant were added to the 30 cm^3^ of alginate solution with glycerol. The solutions were cast onto the Petri dishes and left to solvent evaporate. Water evaporated in surrounding conditions at 23 °C and 40% humidity. The weight ratios of alginate to oil in the films were 2:1 (50%) and 4:1 (25%). Thus, two films containing raspberry seed oil, two with black currant seed oil, and two with both oils were prepared (Figure 5). 

The film thickness measurements were made using a digital gauge (Sylvac GC-050, Yverdon, Switzerland) with an accuracy and resolution of 0.001 mm. The thickness values were the average of several measurements for each sample.

### 4.5. ATR-FTIR Spectroscopy

Infrared spectra of the samples were recorded using the Nicolet iS5 (Thermo Fisher Scientific, Waltham, MA, USA) spectrophotometer equipped with the ATR module (Pike Technologies, Inc., Madison, WI, USA) containing a ZnSe prism of incidence angle 45°. The apparatus settings for collecting spectra were 4 cm^−1^ resolution, 32 scans, and the range of wavenumbers 4000–550 cm^−1^.

### 4.6. UV-VIS Spectroscopy

UV-VIS spectra of the films and solutions were collected using a UV-1601 PC spectrophotometer (Shimadzu, Kyoto, Japan). The UV-VIS spectrophotometer was used for determining antioxidant capacity with the DPPH method. The reduction in DPPH radical was monitored with a UV-VIS spectrophotometer. Moreover, the film opacity was calculated based on UV-VIS spectra of the films. The opacity was calculated as an A_600nm_/film thickness; the thickness was an average value; A_600nm_ was a result read from the UV-VIS spectrum of each film.

### 4.7. The Tensile Tests

The films were stretched using the mechanical testing machine EZ-Test SX Texture Analyzer (Shimadzu, Kyoto, Japan). The samples with the exact shape of paddles were cut out from the films, and then these samples were stretched until rupture with a stretching speed of 10 mm/min. Five repetitions were performed for each film type, and the average values were calculated. The quantity, such as Young’s modulus (E), stress (σ), and strain (ε) at the break, were obtained from the stress–strain curves using the Trapezium X software version 1.4.5 (Shimadzu, Kyoto, Japan).

### 4.8. Moisture Content

The moisture content (Mc, %) in the films was analyzed gravimetrically. After weighing vessels with and without samples, they were dried to a constant weight in an oven at 105 °C. Three repetitions were performed, and the results were averaged. Moisture content was calculated based on the formula:

$$M_{c},\%=\frac{W_{0}-W_{d}}{W_{0}}100\%$$

where W_0_ is the initial sample weight, and W_d_ is the dried sample weight.

### 4.9. Water Vapor Transmission Rate (WVTR)

WVTR was determined using the gravimetric method. EZ-Cups (Berlin, NJ, USA) with a diameter of 63.5 mm (2.5 in.) filled with dried silica gel and covered with the tested samples were weighted each 24 h for seven days. The cups were kept in a desiccator with saturated KCl solution, and the temperature was 23 °C.

### 4.10. Antioxidant Capacity

The samples’ antioxidant capacity (AC) was tested using DPPH (2,2-diphenyl-1-picrylhydrazyl) and applying the QUENCHER method in which the tested films are in direct contact with the DPPH solution [26]. QUENCHER is an abbreviation for QUick Easy New ChEp and Reproducible method [26,36,37]. The studied films are insoluble in the methanol solution of DPPH. The procedure for determining AC was as follows: 0.4 g of the film was ground in a mill and placed in a test tube to which 6 cm^3^ of 60.86 µmol/dm^3^ DPPH solution in methanol was added. The test tube with all its contents was shaken with a shaker (Thermoshaker, VWR International, LLC, Gdańsk, Poland) at 650 rpm for 15 min. Then, the test tube was kept in a dark place for 15 min., after which the UV-VIS spectrum of the supernatant was registered at 517 nm. The results are the average from three measurements for each film, and three series were tested. A formula was applied for the calculation of the scavenging of DPPH as follows:

$$\%DPPH=\frac{A_{0}-A_{s}}{A_{0}}100\%$$

where A_0_ is the absorbance of the initial DPPH solution, and A_s_ is the absorbance of the DPPH solution having contact with the tested film.

Trolox was used to create the calibration curve of the dependence of the percentage of DPPH scavenging (% DPPH) against Trolox concentration. The AC of the tested samples was expressed in μmol of Trolox equivalents per 100 g of tested films [37,38].

### 4.11. Statistical Analysis

Statistical analysis was conducted using ANOVA-one way with Tukey post hoc analysis (*p* < 0.05). Different letters (a–d) within the column mean significant differences between the results.

### 4.12. Antibacterial Activity

The determination of antibacterial activity was carried out based on the standard: PN-EN ISO 20645:2006 “Flat textile products. Determination of antibacterial activity. Agar plate diffusion method” [36]. Standard bacterial strains were used in the research: *Escherichia coli* (ATCC 8739)*, Staphylococcus aureus* (ATCC 6538P)*, Bacillus Subtilis* (ATCC 6051), and *Pseudomonas aeruginosa* (ATCC 13388) (Microbiologist^®^, ST. Cloud, MN, USA).

Agar medium (AM, Oxoid Company, Napean, ON, Canada) containing: tryptone peptone—15 g, phyton peptone—5 g, sodium chloride—5 g, agar-agar—15 g was poured onto Petri dishes and left to gel. Then, the medium was inoculated with a bacterial culture at a concentration of 1.5 × 10^8^ cfu/mL (0.5 McFarland). Tested samples in the shape of a circle with a diameter of 25 ± 5 mm were placed on the dishes. The plates were incubated for 20 h at 37 ± 1 °C. After the incubation time, the ability of the samples to inhibit bacterial growth was determined (inhibition zones, turbidity).

## 5. Conclusions

Active food packaging films were formed of alginate plasticized with glycerol and enriched in RSO and/or BCSO. Introduced oils were responsible for the antioxidant capacity of the films, especially films with raspberry seed oil, which exhibited excellent antioxidant capacity. The presence of oil in films caused an increase in thickness and opacity but a reduction in moisture content and water vapor transmission rate. Moreover, the films containing oil were more flexible and ductile. The infrared spectra showed weak interactions between film components, probably due to the hydrophobic nature of oils and hydrophilic alginate. The films with a higher content of oils were characterized by a higher antioxidant capacity and improved physicochemical properties compared to the sodium alginate film without oil and to films with a lower content of the studied oil. The seeds of common fruits can be a source of active components acquired in the form of oil that can be used in the production of food packaging. 

## Figures and Tables

**Table 1:** Wavenumbers of absorption bands of the studied oils and their assignment to suitable functional groups [10,17,18,19,20,21].

Frequency (cm^−1^)	Vibrations
3008	=C−H stretching in the cis isomers
2923	−C−H asymmetric stretching in CH_2_
2853	−C−H symmetric stretching in CH_2_
1743	−C=O stretching in esters
1705	−C=O stretching in free fatty acids
1650	−HC=CH− stretching in cis isomer
1463	−C−H scissoring in CH_2_, −CH_3_
1377	−C−H scissoring CH_3_
1236	−C−O stretching, −C−H bending
1160	−C−O stretching, −C−H bending
1098	−C−O stretching
964	−HC=CH− bending in trans isomers
914	−HC=CH− bending in cis isomers
721	−HC=CH− bending in cis isomers, C−H rocking −(CH_2_)n−

**Table 2:** Physicochemical properties of the studied sodium alginate films without and with oils.

Sample	Thickness (mm)	Opacity, A_600_/Thickness	M_c_ (%)	WVTR (g/(m^2^ × 24 h))	AC (μmol Trolox/100 g)
Alg + G	0.116 ± 0.009 ^a^	0.436	37.96 ± 3.54 ^a^	296 ± 14 ^a^	0 ^a^
Alg + G+ RSO (25%)	0.166 ± 0.014 ^b^	1.989	32.43 ± 0.96 ^a^	265 ± 2 ^b^	64.71 ± 22.43 ^b^
Alg + G+ RSO (50%)	0.184 ± 0.012 ^c^	2.125	27.94 ± 1.81 ^ab^	245 ± 9 ^b^	128.68 ± 35.92 ^c^
Alg + G + BCSO (25%)	0.134 ± 0.008 ^d^	1.011	26.96 ± 0.15 ^b^	268 ± 3 ^b^	6.78 ± 2.30 ^a^
Alg + G + BCSO (50%)	0.177 ± 0.014 ^c^	1.645	23.40 ± 0.59 ^b^	263 ± 1 ^b^	21.43 ± 3.46 ^ab^
Alg + G + (RSO + BCSO) (25%)	0.136 ± 0.001 ^d^	2.169	27.41 ± 1.44 ^ab^	290 ± 5 ^a^	55.47 ± 0.21 ^b^
Alg + G + (RSO + BCSO) (50%)	0.161 ± 0.008 ^b^	2.466	22.87 ± 1.39 ^b^	253 ± 13 ^b^	59.05 ± 15.22 ^b^

Different letters (a–d) within the column mean significant differences between the results.

**Table 3:** Mechanical properties of the studied films.

Sample	Young’s Modulus, E (MPa)	Stress at the Break, σ (MPa)	Strain at the Break, ε (%)
Alg + G	22.55 ± 3.26 ^a^	7.41 ± 1.18 ^a^	34.75 ± 2.07 ^a^
Alg + G + RSO (25%)	14.68 ± 1.21 ^b^	6.08 ± 1.46 ^a^	41.36 ± 7.83 ^a^
Alg + G + RSO (50%)	11.49 ± 0.69 ^b^	5.57 ± 0.85 ^a^	45.14 ± 4.23 ^ab^
Alg + G + BCSO (25%)	9.12 ± 0.55 ^cb^	5.21 ± 0.66 ^a^	43.84 ± 2.78 ^ab^
Alg + G + BCSO (50%)	7.06 ± 0.67 ^c^	5.24 ± 0.81 ^a^	49.8 ± 2.15 ^b^
Alg + G + (RSO + BCSO) (25%)	12.58 ± 0.56 ^b^	7.15 ± 0.51 ^a^	45.36 ± 2.35 ^ab^
Alg + G + (RSO + BCSO) (50%)	9.8 ± 0.61 ^cb^	4.56 ± 0.55 ^b^	37.79 ± 2.88 ^a^

Different letters (a–c) within the column mean significant differences between the results.

**Table 4:** The acid (AVs), iodine (IVs), and peroxide values (PVs) for raspberry (RSO) and black currant seed oil (BCSO).

Oil Type	AV (mg KOH/1 g Sample)	IV (g I_2_/100 g Sample)	PV (mEq O_2_/kg Sample)	n_D_^25^
RSO	4.03 ± 0.10 ^a^	126.5 ± 4.31 ^a^	20.89 ± 1.31 ^a^	1.4724 ± 0.0005
BCSO	2.65 ± 0.09 ^b^	122.21 ± 3.97 ^a^	21.28 ± 0.95 ^a^	1.4719 ± 0.0005

Different letters (a, b) within the column mean significant differences between the results.

## Data Availability

The data presented in this study are available in the article.

## Supplementary Materials

The following supporting information can be downloaded at: https://www.mdpi.com/article/10.3390/molecules29092012/s1, Figure S1: Growth of bacteria on agar medium with the studied films (left side) and after removing the films (right side).
