# Supplementary material for: Alginate Films Enriched in Raspberry and/or Black Currant Seed Oils as Active Food Packaging
Source: Molecules. 2024 Apr 27;29(9):2012. doi: 10.3390/molecules29092012 (PMC11085223; doi:10.3390/molecules29092012)
Supplement: Supplementary file 1 [file molecules-29-02012-s001.zip › molecules-2942410-supplementary.pdf]

Supplementary Materials

Agar medium with the studied films	Agar medium after removing the films
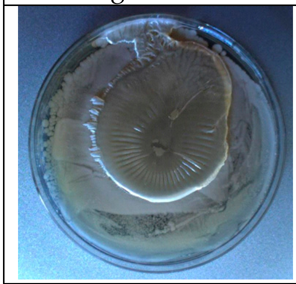	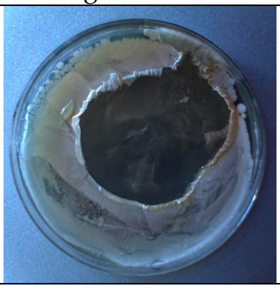
Alg+G+RSO (50%) film on agar with *Bacillus subtilis*	After removing Alg+G+RSO (50%) film from agar with *Bacillus subtilis*
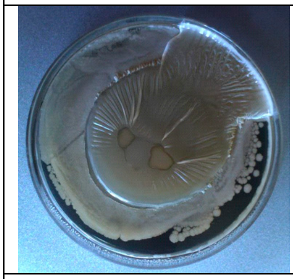	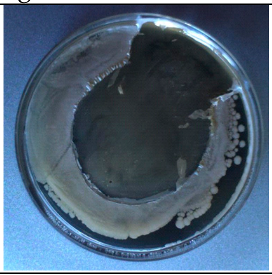
Alg+G+BCSO (50%) film on agar with *Bacillus subtilis*	After removing Alg+G+BCSO (50%) film from agar with *Bacillus subtilis*
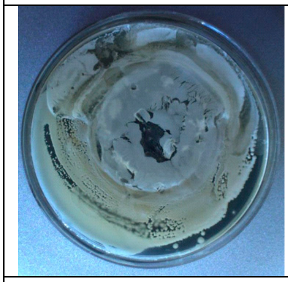	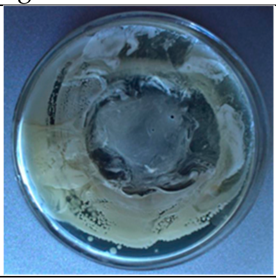
Alg+G+RSO (50%) film on agar with *Pseudomonas aeruginosa*	After removing Alg+G+RSO (50%) film from agar with *Pseudomonas aeruginosa*
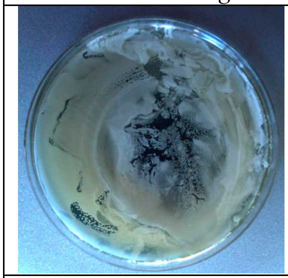	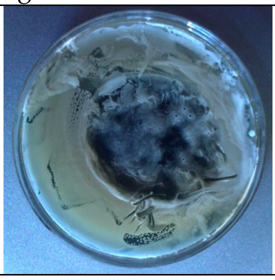
Alg+G+BCSO (50%) film on agar with *Pseudomonas aeruginosa*	After removing Alg+G+BCSO (50%) film from agar with *Pseudomonas aeruginosa*

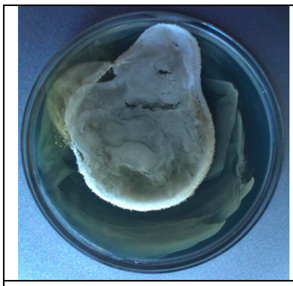	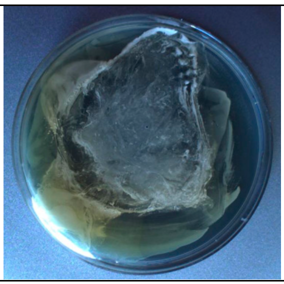
Alg+G+RSO (50%) film on agar with *Staphylococcus aureus*	After removing Alg+G+RSO (50%) film from agar with *Staphylococcus aureus*

Figure. S1. Growth of bacteria on agar medium with the studied films (left side) and after removing the films (right side).
